# Completion pneumonectomy via sternotomy and complete intrathoracic liver migration in metastatic osteosarcoma

**DOI:** 10.1093/jscr/rjad552

**Published:** 2023-10-15

**Authors:** Shantel Chang, Frazer Kirk, Robert Fuller, Sylvio Provenzano

**Affiliations:** Department of Cardiothoracic Surgery, Gold Coast University Hospital, Gold Coast, QLD 4215, Australia; Griffith University, Faculty of Medicine and Dentistry, Gold Coast, QLD 4215, Australia; Department of Cardiothoracic Surgery, Gold Coast University Hospital, Gold Coast, QLD 4215, Australia; Griffith University, Faculty of Medicine and Dentistry, Gold Coast, QLD 4215, Australia; Department of Pathology, Gold Coast University Hospital, Gold Coast, QLD 4215, Australia; Department of Cardiothoracic Surgery, Gold Coast University Hospital, Gold Coast, QLD 4215, Australia

**Keywords:** post-pneumonectomy syndrome, osteosarcoma, sternotomy

## Abstract

Pulmonary metastasectomy is the well-accepted surgical management for recurrent osteosarcoma in the lung. A pneumonectomy is seldom performed, even more so via a sternotomy. We report an unusual case of a pneumonectomy via median sternotomy for a pulmonary metastasis with complete migration of the liver into the intrathoracic space, a complication rarely observed. The patient remains disease-free on follow-up, 21 years following the initial diagnosis. Aggressive approaches for metastasectomy, despite clinician hesitation in the age of minimally invasive surgery, can yield excellent outcomes for a cancer with otherwise poor prognosis.

## Introduction

Pulmonary metastasis is the most common site of spread (90%) for osteosarcoma, usually treated with neoadjuvant chemotherapy and metastasectomy [1]. In a retrospective analysis of pulmonary metastasectomy, majority underwent wedge resections, with no pneumonectomies performed specifically via a sternotomy over 30 years [2]. Herein, we report an unusual case of a pneumonectomy via median sternotomy for removal of pulmonary metastasis, followed by complete migration of the liver into the post-pneumonectomy space

## Case report

**Figure 1:**
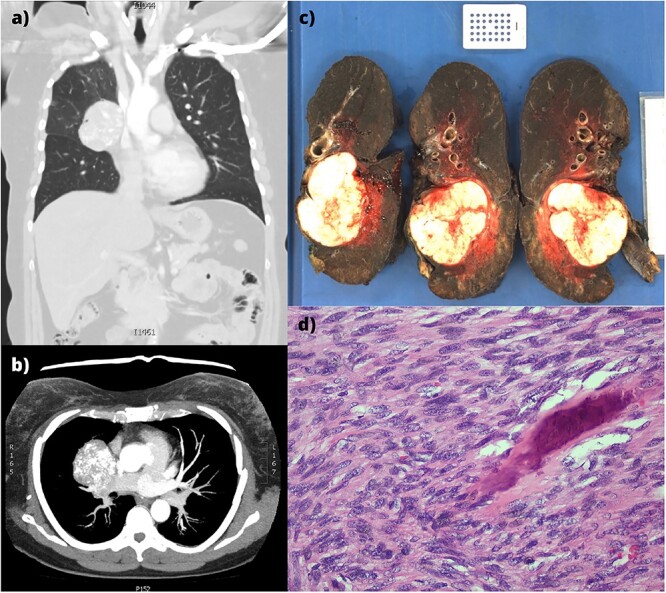
(a,b) CT imaging showing a large right hilar mass with compression of right main bronchus and superior vena cava. (c) Macroscopic appearance of the right lung containing the tumour and adjacent mediastinal structures. (d) Histopathology of the metastatic osteosarcoma, demonstrating atypical spindle cell morphology.

A 58-year-old female with a history of osteosarcoma arising from the right proximal humerus was initially treated with neoadjuvant chemotherapy, resection, and endoprosthetic reconstruction of the humerus, and a pedicled latissimus dorsi flap reconstruction. Four years later, a right middle lobectomy was performed for a metastasis, with histologically clear margins and no nodal involvement. Ten years following the lobectomy, she presented with a new cough. Computed tomography (CT) revealed a right hilar lobulated mass, measuring 7.1 cm × 5.6 cm × 5.9 cm, posteriorly displacing the right main bronchus (Fig. 1a and b). Positron-emission tomography showed significant uptake and an endobronchial ultrasound biopsy confirmed metastatic osteosarcoma.

An *en bloc* intrapericardial right completion pneumonectomy and mediastinal lymph node dissection was performed via median sternotomy. Two factors favoured this approach: the hostile hilum subjected to a previous lobectomy and avoiding injury to the pedicled latissimus dorsi flap used for the initial reconstruction. The lesion was abutting but not involving the superior vena cava. Following lung deflation, the pericardium was opened and the right pulmonary artery, pulmonary vein, and azygous vein were ligated. Station 7 lymph nodes were resected, and the right main bronchus was divided. The right lung, pericardium, phrenic nerve, station 4 and 10 lymph nodes were resected *en bloc* (Fig. 1c). The pericardium was reconstructed with expanded polytetrafluoroethylene (0.6 mm × 10 cm × 15 cm, W. L. Gore & Associates, Inc. Flagstaff, AZ, USA). The soft tissue and sternum were closed routinely.

Histologically, the solitary tumour was well-circumscribed and un-encapsulated with atypical spindle cells in intersecting fascicles (Fig. 1d). Hyperchromasia, pleomorphism, nuclear atypia, and high mitotic indices were demonstrated. Osteoid with calcification was present, confirming osteosarcoma. The bronchovascular margin and lymph nodes were not involved.

**Figure 2:**
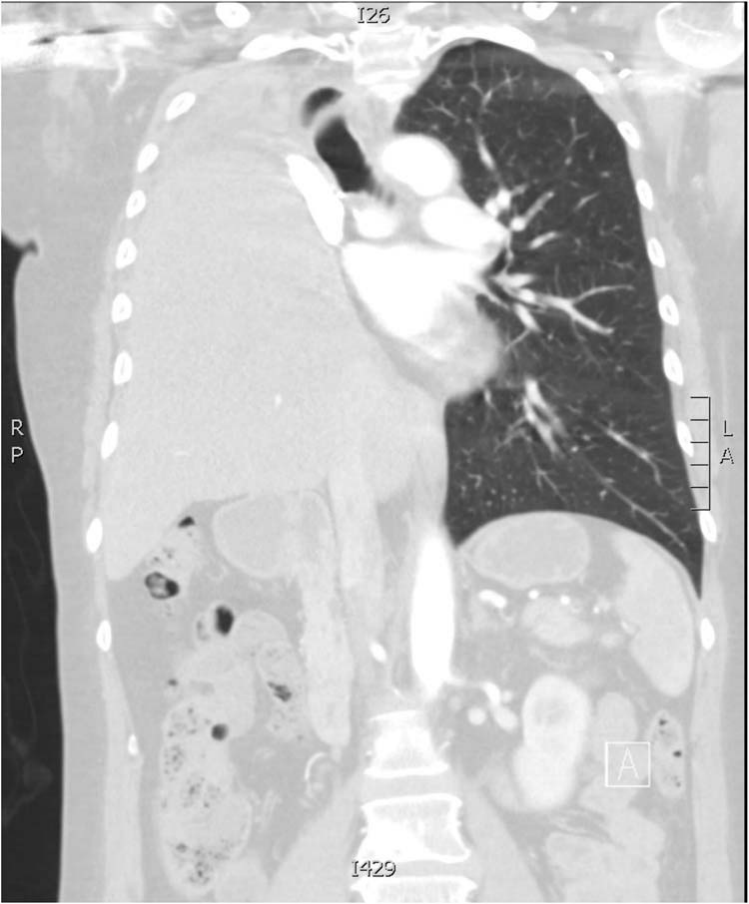
Follow-up CT imaging demonstrating complete migration of the liver into the right thoracic cavity.

The patient had an uneventful post-operative course and subsequently discharged home. Follow-up imaging shows complete elevation of the liver into the right thoracic cavity (Fig. 2). On review 21 years post-initial diagnosis, she remains asymptomatic and disease-free.

## Discussion

The life-expectancy of osteosarcoma patients is 6–11 months from diagnosis without intervention, with 5-year survival rates reported to be 15–64% in the literature [3]. The goal of oncological surgery is complete resection with clear margins while minimizing morbidity. As such, there is a trend towards minimally invasive techniques, with the literature unanimously reporting that wedge resections were most performed, followed by lobectomies. Of 323 patients who underwent metastasectomy over 30 years, only one patient received a pneumonectomy, with an unspecified approach [2]. While a sternotomy is rarely performed for metastasectomy, this case highlights its utility to achieve adequate exposure for large central tumours, a hostile hilum, and when intrapericardial dissection is required.

Post-pneumonectomy hemidiaphragm elevation and mediastinal shift affects the position of vital organs, such as shifting of the heart into the post-pneumonectomy space. A rare post-pneumonectomy syndrome can arise, where there is excessive mediastinal shift resulting in airway obstruction. Iatrogenic harm can be caused, as reported in a case of inadvertent perforation of the right ventricle during blind insertion of a chest tube [4]. In this patient, there is complete migration of the liver into the post-pneumonectomy space, a rarely documented complication. Phrenic nerve resection likely explains the excessive laxity noted in the hemidiaphragm and consequent severe eventration.

## Conclusion

While a pneumonectomy carries significant morbidity and mortality, this approach, in our selected patient, has produced a 21-year survival from diagnosis and exceptional quality of life, for an aggressive cancer with otherwise poor prognosis.

## Data Availability

In the interest of patient confidentiality, no additional data outside of what is disclosed in the article is available.
